# Medical Legal Points

**Published:** 1911-11-18

**Authors:** 


### MEDICAL LEGAL POINTS.

### II.—Death from a Medico-Legal Aspect.

The conditions which hasten the onset of cadaveric rigidity are those which have an exhausting or depressing influence on the muscles immediately before death; hence, after violent muscular exercise, death is speedily followed by rigidity. It has been observed that the bodies of soldiers killed at the commencement of a battle, before they have undergone much fatigue, do not become rigid so soon as those of their fellow-combatants who succumb at a later period after many hours' arduous fighting. After death from diseases or poisons which depress or exhaust the system rigidity comes on early. Animals that have been hunted for some time before death stiffen almost at once, and within a few minutes may be held out by the hind legs perfectly rigid. On the other hand, in speedy death occurring to individuals in vigorous health the onset of cadaveric rigidity is delayed. It may be asserted as a general proposition that the sooner rigidity comes on after death, the sooner will it pass away. The converse is equally true; the longer it is in appearing, the longer will it continue.

When the last attitude of life is maintained after death, considerable light may be thrown on the question whether the case is one of suicide or of homicide. On this ground the precise position in which a body is found demands the careful consideration of the medical jurist. He must in such cases note, therefore, the position of the dead body. Thus, after the suicide of two persons from hydrocyanic acid, the bodies were found firmly folded in each other's arms, as though, after taking the fatal draught, they had embraced one another, died, and stiffened in the act. Again, the suicide may be found sitting in a chair, with dropped arms, but firmly grasping in his hand the pistol, knife, or other instrument employed by him to take away his life. So strong, indeed, under such circumstances may be the grip with which the weapon is held that considerable force may be required to remove it from his hand. Or, again, hand and arm may be fixed in the exact position of the last voluntary act—as, *e.g.*, the attitude assumed in firing a pistol. Or, again, the after-death attitude may betoken the occurrence of a scuffle during life. Thus, from the position and posture of a body we may gather important evidence to help to determine in a case of doubt the question of suicide or homicide. But there are other questions besides this upon which light may be thrown by carefully noting the position and the attitude of a corpse. For example, it is often important to determine whether the place where a body is discovered be the precise spot or not at which the deceased met his death. Or, again, supposing it to be the place, the question may arise whether there is reason to believe that the body has been disturbed or otherwise tampered with since death. But the position of the weapon or the nature of the materials grasped by the hands may constitute evidence of even greater importance than the mere attitude of the corpse. If a weapon likely to have caused the death be found tightly grasped in the hands of a dead body it is important to observe its precise position. Again, any articles found in the hands of a dead body should be carefully preserved. Pieces of dress, for instance, corresponding to that worn by a suspected person; or hair corresponding to that of the accused, clutched during a scuffle between him and his victim—have many times constituted evidence of the greatest value. Again, the force with which the various articles are grasped should be carefully and immediately noted. Immediately, because when once the articles have been removed from the hands no conclusions of any value can be formed by after experiments or observations (the conditions being so materially disturbed by handling) as to the power with which they were grasped.

The following points are worth attention: If a weapon be found loosely held in the hands of the deceased, no conclusion of value can be deduced as to the question of suicide or homicide. But if a weapon be found firmly grasped by the deceased person, suicide rather than homicide is indicated. It would be difficult for a murderer to place a weapon in the hands of his victim after death, unless the muscles were warm and pliant at the time; and this being the case, it is scarcely possible to conceive that the instrument would be found tightly grasped unless it was held or secured in position during the period of muscular relaxation, and until post-mortem rigidity supervened. But it is doubtful how far even this would be successful.

### Putrefaction.

The last of the phenomena which under ordinary conditions follows death, is putrefaction, or the resolution of the organised tissues into their elementary constituents. Complex organic bodies step by step are resolved into simpler forms, until ultimately they are split up into inorganic substances. Putrefaction is the only absolute sign that death has taken place. The agencies concerned in the causation of putrefaction are micro-organisms, moisture, air, and warmth. After a longer or shorter time the whole organic mass is changed to inorganic matter, chiefly water, ammonia, and carbonic acid, and in the transition stage compounds of nitrogen, sulphur, carbon, and hydrogen are evolved.

The bodies of persons who have died from acute diseases have been observed to putrefy more readily than those of persons who have died from wasting and chronic disease. Some poisons, by chemically combining with animal matter, appear to confer on it the power of resisting putrefaction—at least to a very great degree. This is now a well-known property of arsenic, and in the arts this poison is largely employed as an antiseptic. When a solution of it is injected into the arteries of a dead body, it tends to preserve it for a long time from putrefaction. In examining the bodies of persons poisoned by arsenic after an interment of six, twelve, or twenty-four months it has been found that the stomach and bowels were remarkably preserved, and the liver, spleen, and heart also preserved, but in a less perfect manner.

### General Inferences to be Drawn.

The postures in which the bodies of persons found dead from any cause are discovered may, in numerous cases, be brought forward to support a charge of murder, or at least of criminal interference. When a body is found rigid with the members evenly extended and the jaws closed, this is *cœteris paribus*, strongly indicative of interference while there was warmth and pliancy in the limbs. When, on the other hand, the body is found rigid and doubled up, with the limbs more or less twisted, lying on an even surface like a bed, the probability is, according to circumstances, that the body had been moved from the spot in which the person had died, and in which rigidity had supervened.

The changes which take place in a dead body before the commencement of putrefaction, if accurately observed, may sometimes enable a medical witness to form an opinion of the time at which the deceased died. The dead body of a person may be found in a house with marks of murderous violence upon it. The crime may have been so recently perpetrated that the body still retains the warmth and pliancy observed in the recently dead, or it may be found in a cold and rigid state. A person charged with the murder may be able to prove that he had not been in the house for many hours or days, or evidence may be adduced to show that he was there at a time which would correspond to the condition of the body when found. In cases of sudden death from violence or suspected poisoning, a medical man by observing the state of the body may frequently form a judgment of the time at which death occurred, and therefore of the period at which poison was taken by deceased, or violence was inflicted on the body. In forming a judgment of priority of death in cases of murder and suicide, the sufficiency of the wound to produce instant or rapid death must always be taken into consideration. A person may inflict on another a slight wound which may prove fatal by hæmorrhage only after some hours, while he may afterwards inflict upon himself a wound which would instantly destroy life. In such a case the body of a murderer would be found cold, while that of the victim, by reason of the death being more recent, would be warm.

There is great difficulty in the practical application of the principles laid down to the determination of the probable period of death in reference to the body of a person found dead. Medical opinions are frequently required in cases of alleged child-murder or the probable date of death of a child whose body has been found in a putrefied state. With a due regard to the conditions under which the body has been placed, a medical witness may occasionally be able to say whether its state was such as to be consistent or inconsistent with the delivery of an accused woman at a particular period of time. Greater difficulties occur on charges of murder in reference to the bodies of adults found dead under suspicious circumstances. The connection of an accused person with the act may occasionally turn upon a medical opinion respecting the state of the dead body.

In the case of a body found in the water, death may obviously have happened prior to immersion from natural causes or from some form of violent death, such as hanging or strangulation, which gives rise to the characteristic appearances of asphyxia, so that we have to consider whether the post-mortem appearances alleged to be characteristic of death by drowning might have been occasioned by causes acting before immersion, and whether, in the case of bodies which have remained in the water some time, the appearances usually attributed to the mode of death may not be explained by the circumstances of the immersion itself.

With regard to injuries on the bodies of persons found in the water, three questions arise: (1) Were they inflicted during life? (2) If so, are they such as to account for death before submersion? (3) Were they accidental, suicidal, or homicidal? There are five ways in which a body taken from the water may come to exhibit marks of violence: (1) A man may be murdered (by poison or other means) and thrown into the water dead; (2) he may receive injuries from the hands of others or himself, and may then be thrown (or throw himself) into the water while still alive. If the injuries are in the shape of bruises they may have been caused—(3) by the death struggles; (4) by some obstacle against which the body is borne; (5) in the very act of falling into the water.

In a man who has been murdered and thrown into the water dead, we should expect to find all the signs of death by drowning absent, with the exception of such as may have been caused by uncommon depth of water or advanced putrefaction. Dislocation of the limbs is a possible consequence of falling from a great height. The presence of water in the stomach is deemed by Orfila to be the most satisfactory proof we have of drowning during life.

There is but little difference between the specific gravity of the human body and that of water, though the former is somewhat the greater. Hence a person, whether dead or alive, when thrown into the water will sink, unless buoyed up by external aid; but after the process of putrefaction has occasioned the evolution of gaseous matter, the body becomes specifically lighter than the water, and it rises to the surface. It is for this reason that bodies committed to the deep have generally weights affixed to them. It is, however, possible that a body may float at first when its cavities have been filled with air. Thus Dr. Male supposes that in the case of a person strangled and thrown into the water with the cord attached around the neck, the body might float at once from the included air. The body of Prince Carraccioli, who was hanged by order of Lord Nelson, was sunk into the sea with double-headed shot weighing 250 lb. tied to the legs. It floated on the surface in thirteen days. (Male, p. 115.)

